# Exercise cardiac MR assessment of diastolic function

**DOI:** 10.1186/1532-429X-17-S1-P26

**Published:** 2015-02-03

**Authors:** Christopher J Francois, Omid Forouzan, Jared Warczytowa, Jacob A Macdonald, Oliver Wieben, Naomi C Chesler

**Affiliations:** 1 Radiology, University of Wisconsin - Madison, Madison, WI, USA; 2 Biomedical Engineering, University of Wisconsin - Madison, Madison, WI, USA; 3 Medical Physics, University of Wisconsin - Madison, Madison, WI, USA

## Background

Dyspnea with exertion is a common symptom in patients with left ventricular (LV) systolic and diastolic dysfunction. Assessing changes in systolic and diastolic hemodynamic parameters with exercise is necessary to thoroughly characterize these patients. Evaluation of changes in LV systolic function with exercise stress cardiac magnetic resonance (MR) has been demonstrated previously [1,2]. In this study we assessed the feasibility of assessing LV diastolic function with exercise cardiac magnetic resonance.

## Methods

14 healthy subjects (26.1±4.7 years, 5 men/9 women) were prospectively recruited according to an IRB-approved and HIPAA-compliant protocol. Supine, exercise cardiac MR was performed on a 1.5T scanner (HDx and 450W, GE Healthcare, Waukesha, WI) using an MRI-compatible exercise device that enables exercise to be performed on the scanner table [3]. Transmitral inflow was assessed with 2D phase-contrast (PC) MRI (FOV=370x260mm^2^; matrix=256x128; TR/TE=6.1/3.7ms; FA=30°; ASSET=2; VENC=100cm/s) acquired through the tips of the MV leaflets during diastole [4]. Exercise was performed at a constant workload (36.1±7.5W) for >3 minutes. The flow measurements were acquired during a breath-hold immediately following cessation of exercise to minimize bulk motion artifacts. 2D PC MR images were analyzed with CV Flow (Version 3.3, Medis, Leiden, the Netherlands). Resting and exercise E and A velocities and E/A ratios were recorded for each subject from the transmitral inflow-time curves. The paired Student's t-test was used to determine if differences between exercise and baseline were statistically significant.

## Results

Exercise MV flow data was successfully acquired in 12/14 subjects with heart rates increasing 25±10.6bpm relative to rest. In 2/14 subjects, motion artifacts rendered the images unusable for analysis. E and A velocities and E/A ratios were higher than at rest, although the differences were not significant for the entire cohort (Table 1). In subjects that had an increase in heart rate >20bpm, E-velocities did increase significantly.

**Table 1:** Summary of E- and A- velocities and E/A ratios at rest and following exercise.

Subjects	Condition	E (cm/s)	A (cm/s)	E/A
All (N=12)	Rest	82.0±18.7 (61.6-134.4)	53.0±17.3 (34.2-95.6)	1.63±0.43 (1.00-2.35)
	
	Exercise	89.1±6.7 (76.5-98.3) P=0.25	56.5±16.1 (32.4-94.5) P=0.63	1.69±0.45 (0.81-2.50) P=0.73

<20bpm increase in HR (N=5)	Rest	92.7±24.7 (68.4-134.4)	55.9±17.7 (42.1-83.6)	1.74±0.50 (1.00-2.12)
	
	Exercise	88.2±6.1 (79.8-95.3) P=0.74	53.3±13.7 (32.4-66.1) P=0.75	1.74±0.43 (1.41-2.46) P=0.99

>20bpm increase in HR (N=7)	Rest	73.6±11.8 (61.6-95.6)	51.1±20.7 (34.2-95.6)	1.56±0.45 (1.00-2.35)
	
	Exericse	89.8±7.4 (76.5-98.3) P=0.01	58.8±18.4 (35.4-94.5) P=0.50	1.66±0.50 (0.81-2.50) P=0.71

Values are reported as mean ± standard deviation (range). There were trends of higher E- and A- velocities and E/A ratios, but the increase in E-velocity was only significant in subjects in whom heart rate increased >20bpm relative to rest.

## Conclusions

Quantification of exercise stress transmitral flow with MRI was feasible in the majority of healthy subjects, enabling the evaluation of exercise-induced changes in diastolic function. The findings of higher E and A indices is concordant with previously published data using exercise-stress echocardiography [5,6].

## Funding

NIH R01HL105598.

**Figure 1:**
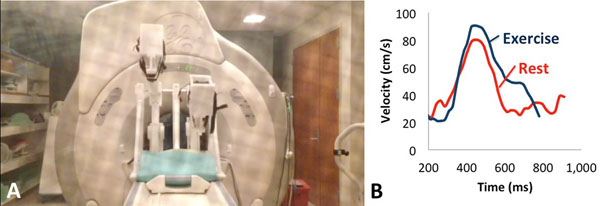
(A) MRI-exercise device with volunteer exercising in scanner. (B) Mitral valve flow-time curves at rest and during exercise in a healthy subject. The E/A ratios decreased and the E-wave deceleration times increased with exercise.
